# Bridging the Gap: Time to Integrate Sex- and Gender-Specific Insights into Research and Practice

**DOI:** 10.1007/s12471-025-02002-w

**Published:** 2025-11-11

**Authors:** Lotte Paulis, Maryam Kavousi

**Affiliations:** Department of Epidemiology, Erasmus MC, University Medical Center, Rotterdam, The Netherlands

Despite extensive public health campaigns, cardiovascular disease (CVD) remains the leading cause of death among women worldwide, with women still being underdiagnosed, undertreated, and underrepresented in cardiovascular research [1]. To address this gap, this issue features six articles that examine the complex interplay of sex-specific pathophysiology and gender-related determinants in CVD, providing insights for research, clinical practice, and policy, and highlighting ongoing initiatives within the Netherlands’ research landscape.

Sex refers to biological attributes, such as anatomy, gene expression, and hormone profiles, which classify individuals as female, male, or intersex [2]. While closely intertwined, sex differs from gender. Gender reflects the socially constructed roles, behaviors, and norms attributed to women, men, girls, boys, or gender diverse individuals, and these constructs evolve across cultures and over time [2]. Both sex and gender shape health outcomes: sex influences biological pathways and pathophysiological processes underlying diseases, whereas gender affects illness perception, health-seeking behavior, risk exposure, psychosocial stress, and treatment responses [2]. Gender equality is not solely a concern for women; it also involves men, whose socially reinforced risk-seeking behaviors, involvement in violence, and barriers to discussing mental health problems have important implications for their own health and that of women, underscoring the need to include men in this conversation.

Optimizing cardiovascular health in women requires a sex-specific, life-course approach to cardiovascular prevention and management. From menarche to menopause, women undergo profound hormonal and metabolic transitions that shape their cardiovascular health. The review by Siddiqui et al. highlights that female-specific and female-predominant risk-enhancing conditions, such as pregnancy complications, gynecological disorders, and autoimmune diseases, are often overlooked in existing risk assessment tools for CVD [3]. Concurrently, traditional risk factors, including hypertension, diabetes, hypercholesterolemia, obesity, and smoking, tend to exert a greater impact on women [3]. Furthermore, Siddiqui et al. report sex-specific variations in blood pressure and cardiac biomarkers, while Freriks et al. highlight electrophysiological differences between men and women—areas that remain under-researched and insufficiently integrated into clinical practice [3, 4]. Recognizing female-specific risk factors and thresholds enhances cardiovascular management of women by enabling early detection of high-risk individuals, initiating timely preventive interventions, and reducing diagnostic delays.

Gender-related determinants, such as socioeconomic disadvantage, unequal access to healthcare, caregiving responsibilities, occupational stress, psychosocial stress, intimate partner violence, and gender-based discrimination, further amplify cardiovascular risk and hinder prevention, diagnosis, and treatment among women [3]. The impact of sex- and gender-related risk factors is particularly pronounced in non-white women, underscoring the influence of ethnicity and sociocultural context on cardiovascular risk, and highlighting the need for a broad, inclusive perspective as noted by Dal Canto et al. [5]. Collectively, these gaps underscore the urgent need to integrate sex- and gender-specific determinants of cardiovascular health into research, clinical guidelines, and everyday practice.

Achieving sex equality in cardiovascular care requires greater inclusion of women in clinical trials and focused research on conditions that predominantly affect women. For instance, disorders disproportionately affecting younger women—such as angina with non-obstructive coronary arteries, as discussed by Dal Canto et al., and spontaneous coronary artery dissection, as reviewed by Kalkman et al.—remain under-investigated [5, 6]. The current clinical practice relies on diagnostic tools developed for obstructive coronary artery disease, contributing to delays in diagnosis and uncertainties in the treatment of women. Dal Canto et al. reviewed the Dutch research landscape and highlighted initiatives designed to address knowledge gaps in these conditions by promoting tailored diagnostic strategies and raising awareness to improve timely recognition and management [5].

Additionally, research efforts in the Netherlands have increasingly focused on understanding the causes of women’s underrepresentation in clinical trials and assessing their impact on treatment outcomes [5]. The persistent underrepresentation of women in cardiovascular research challenges the validity of current diagnostic criteria, risk prediction tools, and treatment strategies. When the foundation of evidence-based medicine is built predominantly on male populations, it raises uncertainty about how confidently these findings can be applied to women—and even more so to women from diverse ethnic backgrounds. Encouragingly, van der Bijl et al. suggest that the efficacy of cardiovascular therapies may be comparable between sexes, while Li et al. report that diagnostic algorithms for heart failure perform similarly in men and women [7, 8].

However, van der Bijl et al. emphasize that efficacy demonstrated in controlled trials does not necessarily translate into real-world benefits [7]. Women experience higher rates of adverse drug reactions, which may lead to lower adherence and poorer outcomes [7]. Women enrolled in clinical trials may not fully represent the broader population of women with CVD, potentially leading to real-world outcomes that differ from those observed in clinical studies. Thus, van der Bijl et al. argue for inclusive trial designs and systematic, sex-specific safety reporting to advance both accuracy and equality in cardiovascular care [7].

Closing the cardiovascular health gap for women requires more than awareness; it demands structural changes in research and clinical guidelines. Achieving this will require inclusive trial designs at all phases, systematic sex-specific safety reporting, and focused attention on conditions that disproportionately affect women. Equally important is ensuring representation of women from diverse ethnic and socioeconomic backgrounds. Lastly, integrating the intertwined influences of sex and gender into diagnosis, prevention, and treatment strategies is essential to improve cardiovascular outcomes for women worldwide.
